# Mass spectrometry imaging in gynecological cancers: the best is yet to come

**DOI:** 10.1186/s12935-022-02832-3

**Published:** 2022-12-19

**Authors:** Dagmara Pietkiewicz, Szymon Plewa, Mikołaj Zaborowski, Timothy J. Garrett, Eliza Matuszewska, Zenon J. Kokot, Jan Matysiak

**Affiliations:** 1 Department of Inorganic and Analytical Chemistry, Poznan University of Medical Sciences, 3 Rokietnicka Street, 60-806 Poznan, Poland; 2 Gynecologic Oncology Department, Poznan University of Medical Sciences, 33 Polna Street, 60-535 Poznan, Poland; 3 Institute of Bioorganic Chemistry, Polish Academy of Sciences, Poznan, Poland; 4 Department of Pathology, Immunology, and Laboratory Medicine, University of Florida, Gainesville, FL 32610 USA; 5 Faculty of Health Sciences, Calisia University, 13 Kaszubska Street, 62-800 Kalisz, Poland

**Keywords:** Mass spectrometry imaging, Cancer research, Ovarian cancer, Endometrial cancer, Vulvar cancer

## Abstract

Mass spectrometry imaging (MSI) enables obtaining multidimensional results simultaneously in a single run, including regiospecificity and *m/z* values corresponding with specific proteins, peptides, lipids, etc. The knowledge obtained in this way allows for a multifaceted analysis of the studied issue, e.g., the specificity of the neoplastic process and the search for new therapeutic targets. Despite the enormous possibilities, this relatively new technique in many aspects still requires the development or standardization of analytical protocols (from collecting biological material, through sample preparation, analysis, and data collection, to data processing). The introduction of standardized protocols for MSI studies, with its current potential to extend diagnostic and prognostic capabilities, can revolutionize clinical pathology. As far as identifying ovarian cancer subtypes can be challenging, especially in poorly differentiated tumors, developing MSI-based algorithms may enhance determining prognosis and tumor staging without the need for extensive surgery and optimize the choice of subsequent therapy. MSI might bring new solutions in predicting response to treatment in patients with endometrial cancer. Therefore, MSI may help to revolutionize the future of gynecological oncology in terms of diagnostics, treatment, and predicting the response to therapy. This review will encompass several aspects, e.g., contemporary discoveries in gynecological cancer research utilizing MSI, indicates current challenges, and future perspectives on MSI.

## Introduction

**Fig. 1:**
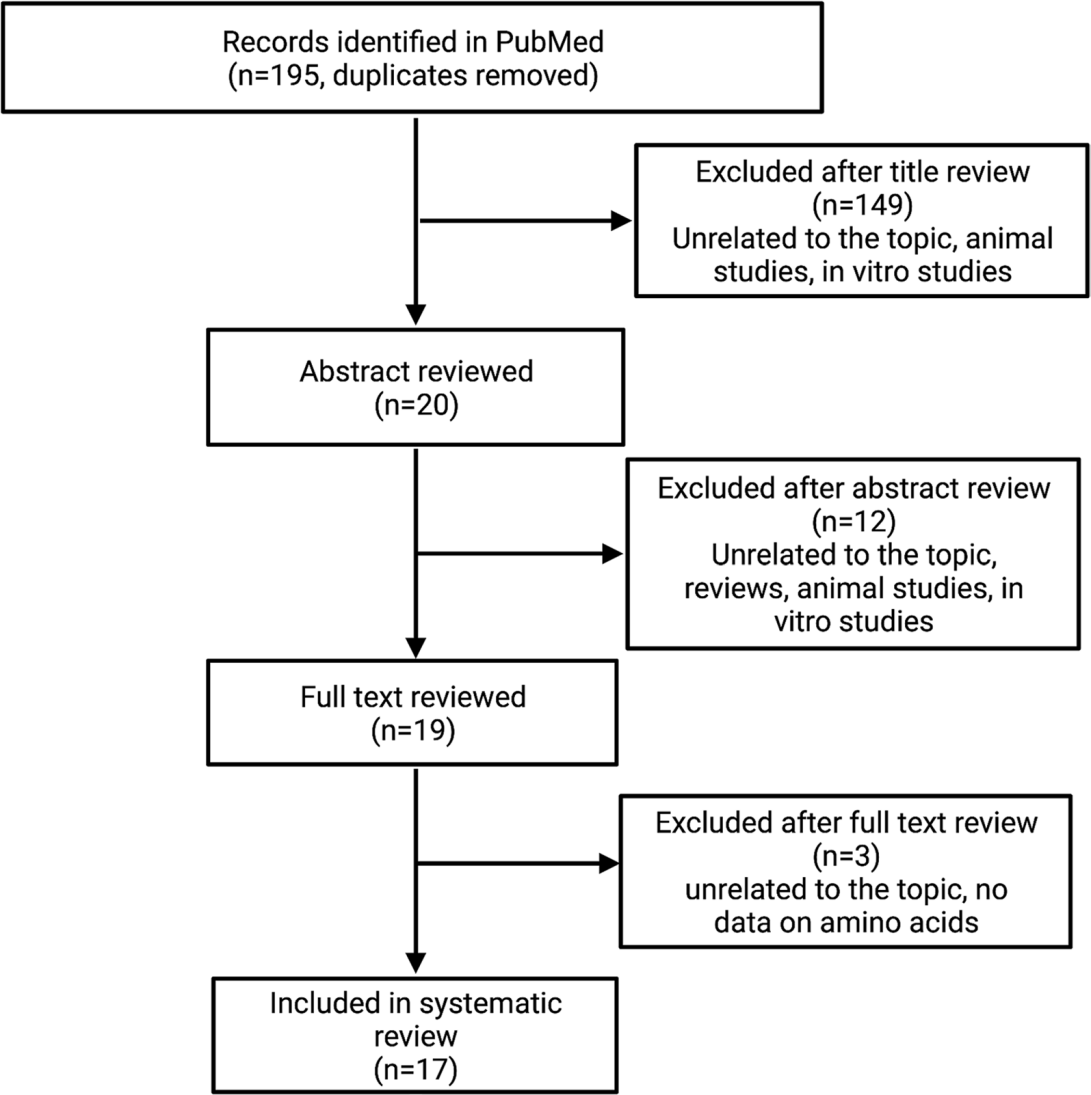
Algorithm of the literature search for reviewed articles. Created in BioRender

Despite the significant advance, gynecologic malignancies, especially ovarian cancer (OC), remain associated with poor prognosis [1]. The detailed studies of gene expression at the transcriptome and proteome levels have contributed to the improved detection and treatment of gynecological cancers [2–6]. Most women who have OC develop therapy resistance. Patients with recurrent platinum-resistant OC have poor survival and limited treatment options that have not yet been standardized. Therapies targeting DNA repair pathways have demonstrated a favorable safety profile and promising results [7, 8]. Poly (ADP-Ribose) Polymerase inhibitors (PARPi), such as niraparib or olaparib, have resulted in a beneficial overall response rate as compared to other standards of care [8]. Another PARPi–niraparib was effective in combination with an anti-PD-1 antibody, pembrolizumab [7] or with bevacizumab [9, 10]. Consistently, all these studies demonstrate narrow groups of patients that respond to novel treatment options. However, it remains unclear which patients can benefit from given combination therapy. Therefore, a search for novel techniques to analyze tumor biology is necessary to optimize management and indicate new therapy targets. MSI has the potential to bring another new dimension to the description of cancer phenotypes and identify biomarkers of response to novel therapies (see Fig. 1).

## Literature search

**Fig. 2:**
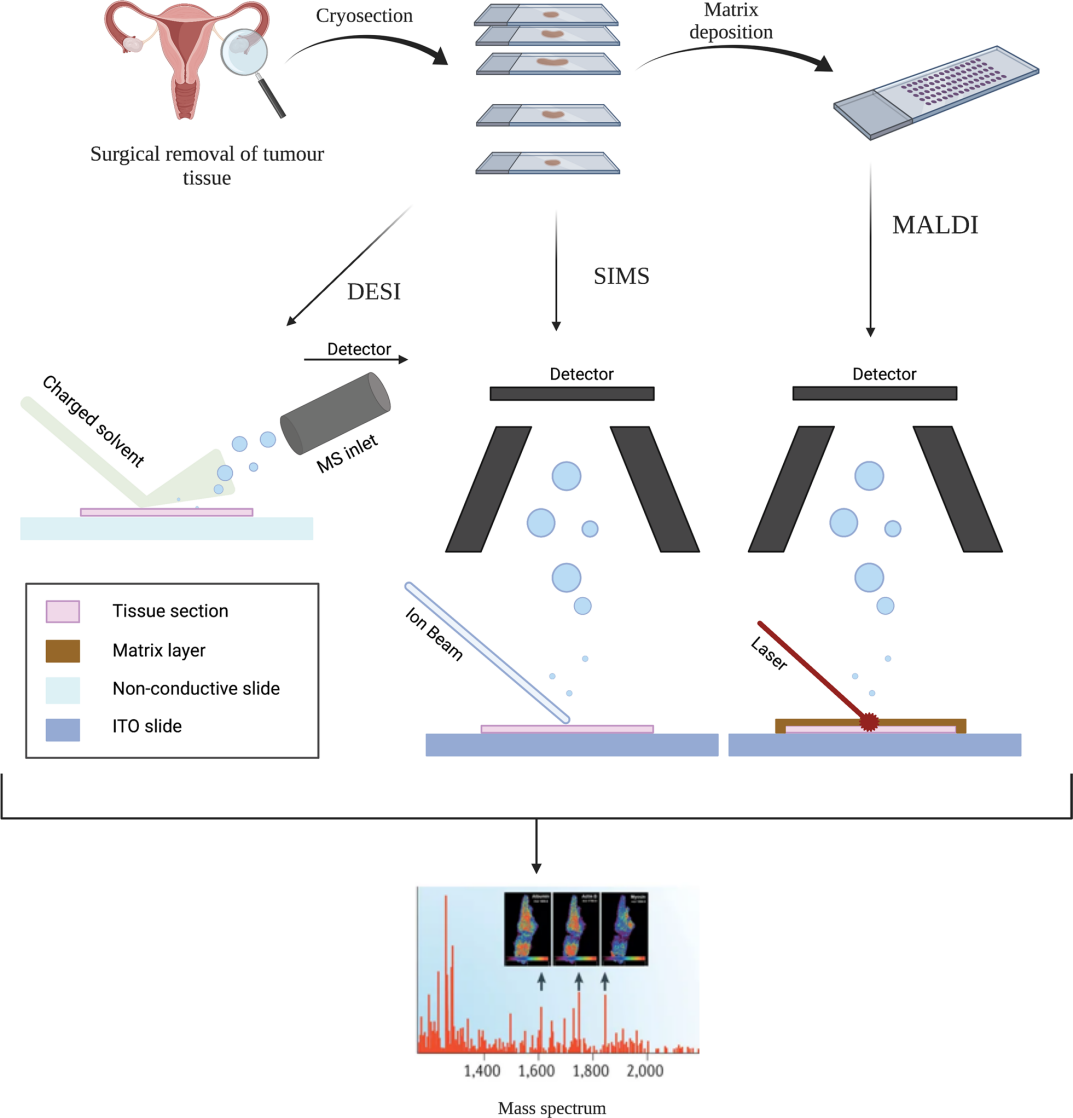
Principles of MSI workflow for the most used ionization approaches for tissue imaging. Created in BioRender

A systematic, comprehensive literature search of studies published from January 2011 to January 2022 was conducted using the PubMed database. This timeframe was chosen to ensure the inclusion of articles relevant to contemporary healthcare. The search was conducted using medical subject headings (MeSH) search terms. We used combinations of the following keywords: ‘endometrial cancer’, ‘ovarian cancer’, ‘epithelial ovarian cancer’, ‘cervical cancer’, ‘chorionic cancer’, ‘choriocarcinoma’, ‘vulvar squamous cell carcinoma’, ‘vulvar cancer’, ‘mass spectrometry imaging’, ‘MALDI-MSI’, ‘DESI-MSI’, ‘MALDI mass spectrometry imaging’, ‘DESI mass spectrometry imaging’. Studies focused on the application of mass spectrometry imaging methods in gynecological cancers research were included in this review. Cohort studies, case studies, and technical briefs were excluded. The articles retrieved through the search of the database were screened by one author. Firstly, all studies were screened based on the title and then based on the abstract to identify the papers that met the inclusion criteria. The screening process was followed for the full-text review by two authors. The results of the performed screening are presented in Fig. 2.

## Present applications of mass spectrometry imaging

MSI plays an important role as a diagnostic and prognostic tool in cancer. This technique generates label-free mass spectra providing nonbiased, nontargeted molecular information. MSI has a variety of applications, e.g., biomarker discovery [11], tissue classification [12], molecular histology [13], and drug imaging [14]. Using MSI, we can distinguish tissue types [15], determine tumor margins [16], predict metastasis [17], analyze chemoresponse [18], and identify diagnostic and prognostic markers [19, 20]. Taken together, MSI has a chance to become a powerful tool in clinical routine.

### Biomarker discovery

**Fig. 3:**
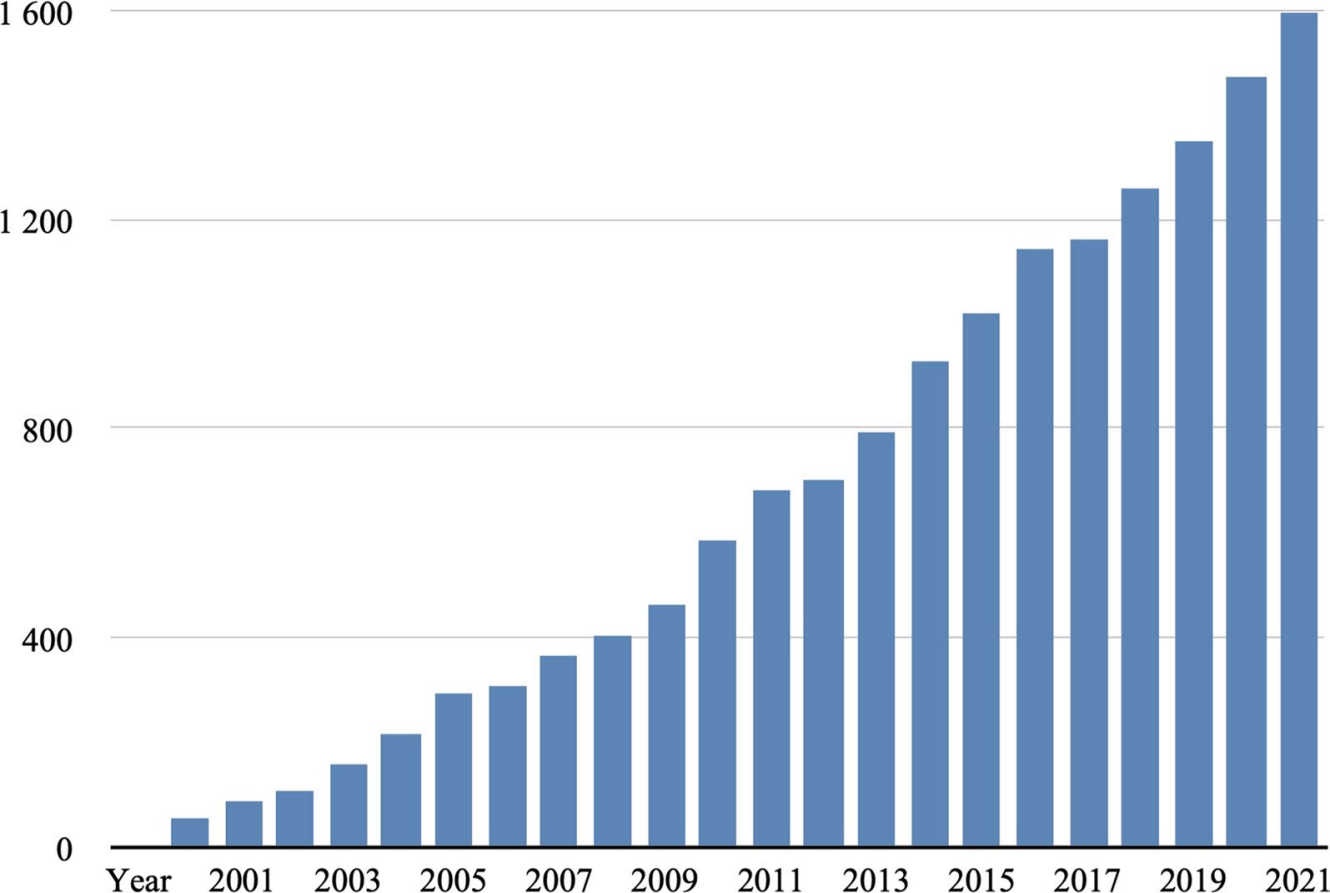
Number of papers published per year on mass spectrometry imaging. Note: The search engine PubMed was used with the keyword ‘mass spectrometry imaging’. From www.pubmed.com

Clinical decisions are made based on laboratory test results [21]. Various technologies enable the observation of the changing chemical composition of human body fluids and, on that ground, identify potential biomarkers for detecting, predicting, and monitoring a disease or drug treatment. A biomarker’s utility is designated by its diagnostic sensitivity and specificity [22]. Diagnostic sensitivity is the probability that the diagnostic test will be positive when the disease is present. Diagnostic specificity is the likelihood that the diagnostic test will be negative when a patient does not have the disease. High sensitivity for screening markers is important to not miss any cancers, but high specificity reduces the potential costs of the follow-up testing to confirm the diagnosis based just on the biomarker test result. Identification of the biomarker starts with measurable change, most likely in the downstream components of molecular cascades that may be investigated not only from initiation but from the accompanying events that may lead to potential biomarker candidates. An ideal biomarker is characterized by easy sampling (collected in a minimally invasive manner), simple and reliable measuring, and is clearly correlated with the outcome [23]. Proteomic-based research provides insight into protein composition and functions. For investigating global protein composition, tissue extracts or homogenized organs are analyzed. However, changes originating at the cellular level might not be captured by solution-based techniques due to the lack of sensitivity. Tissues have the additional advantage that the tumor microenvironment can also be considered, containing a high number of proteins for the discovery of new potential biomarkers. MSI is an advanced technology enabling visualization of molecular changes at the cellular level without losing important histological information about the molecules’ distribution. This technique couples traditional histological approaches with advanced mass spectrometry. The most popular ionization approaches are desorption electrospray ionization (DESI) [24, 25], matrix-assisted laser/desorption ionization (MALDI) [26, 27], and secondary ion mass spectrometry (SIMS) [28] as they are commonly used for tissue imaging (Fig. 2). Other approaches serve advantages, such as a laser ablation electrospray ionization [29], liquid microinjunction sampling [30], and matrix-assisted laser desorption electrospray ionization (MALDESI) [31]. MSI is becoming widely accepted, as shown by the increasing number of publications in recent years (Fig. 3). Many studies have shown the strength of this emerging technology in biomarker research [26, 32, 33] as MSI has the capability to overcome some of the limitations present in other approaches like the loss of crucial histological information and significant differences that might be observed only as a localized histological target concentration, the lack of possibility to correlate the results with traditional histology, not being able to address many varied analytes on a single platform. A key next step for the MSI technique is its translation to effective applications in the clinical laboratory.

### Tissue classification

Many gynecological cancers are heterogeneous tumors. Each with its own histological subtypes, different morphology, molecular biology, and clinical outcome. That means that personalized medicine is a top priority in modern oncology and without proper tissue classification it is of limited use. In clinical studies involving histological review of the tumor, misdiagnosis is not uncommon and can be as high as 15% [34]. Gene expression profiling is one of the approaches for looking at new therapeutic targets. In the case of high-grade serous OC (HGSOC) Kassuhn et al. [27] proposed the classification of molecular subtypes of HGSOC by MALDI-Imaging supported by machine learning. They performed MALDI-MSI analysis along with RNA extraction and NanoString classification. The results of the analyses performed indicated that the molecular background responsible for HGSOC subtypes and clinical diversity is visible on both the gene expression and proteomic level. They implemented MALDI-MSI for the classification of the stroma compartment of tissue cores. It turned out that this approach provided near-perfect stroma classification.

Thus, MSI seems to be a promising tool in tissue classification, providing a chance for personalized medicine to be a clinical laboratory procedure. However, more research needs to be conducted to draw firm conclusions about the usefulness, reproducibility, and reliability of MSI.

### Molecular histology

Currently, we are in the era of multiomics, where classical histology is more often overlaid with molecular profiles (i.e., lipidomic, metabolomic, proteomic) obtained by MSI. With the progression of cancer, the inherent heterogeneity of the tissue increases. Thus, the need of introducing mass spectrometry techniques that are label-free and can visualize the spatial distribution of molecules within the analyzed tissue section is increasing. MSI-based molecular histology is more often used in oncology research [35]. One of the most often used techniques is MALDI-MSI. Higher speed, higher resolution, and higher sensitivity make MALDI-MSI a valuable tool in biomarker discovery research. MALDI-MSI is not restricted to the analysis of a single protein (like IHC), so the images of the spatial distribution from a broad variety of biomolecules can be generated. Introducing MSI into the clinical laboratory routine would enable molecular characterization of tumors for each patient individually which would translate into personalized cancer treatment. MALDI-MSI has been applied in a variety of cancer research [36–39]. One of the main concerns with introducing MSI into clinical pathology was the uncertainty of whether the IHC staining is feasible on tissue sections after MSI analysis. Mittal et al. [40] compared predictive models of metastasis in endometrial cancer patients. Models were generated from IHC and MALDI-MSI analyses. When they compared the two models, MALDI-MSI showed higher prognostic accuracy than the IHC model.

## Mass spectrometry imaging in gynecological cancers

Gynecological cancers are highly prevalent among women worldwide. Current histopathological diagnoses are based on the subjective interpretation of, for example, immunohistochemical staining. The ability to correlate the results of MSI analysis with histological features provides information that supplements conventional histological classification with more clinical information. An overview of mass spectrometry imaging studies was presented in Table 1.

**Table 1:** An overview of mass spectrometry imaging studies on ovarian, endometrial, and vulvar cancer and their results

Authors	Groups	Technique	Input material	Conclusions of the study
Ovarian cancer				
Wanja Kassuhn et al. [27]	HGSOC tissue samples (n = 279)	MALDI-MSI, nanoLC-MS/MS	FFPE	Selection of 135 peptides able to classify HGSOC subtypes (MALDI-derived predictive proteomic signature). Identification of 91 of these peptides as 56 proteins
Dagmara Pietkiewicz et al. [41]	Low-grade serous borderline ovarian tumor (n = 1) and ovarian fibrothecoma (n = 1) tissue samples	MALDI-MSI	FF	Demonstration of the potential of the MALDI-MSI technique by showing regiospecific m/z values to improve the diagnosis of ovarian tumors, particularly in the most challenging cases
Hua Zhang et al. [42]	Human laryngeal cancer tissue sample (n = 1) and ovarian cancer tissue sample (n = 1)	MALDI-LTQ-Orbitrap-MS	FFPE	Demonstration of the usefulness of the on-tissue labelling strategy coupled with MALDI-MSI for the sensitive spatial characterization of N-glycan expression within heterogeneous tissue samples
Matthew T. Briggs et al. [26]	FIGO stage I (n = 3), and stage III (n = 3) serous ovarian cancer tissue samples	MALDI-MSI, PGC-LC–MS/MS	FFPE	Characterization of spatial distribution across tumor and non-tumor regions of 14 N‐glycans by MALDI‐MSI. Identification and structural characterization of 42 N‐glycans (including structural and compositional isomers) by LC–MS
Arun V. Everest-Dass et al. [43]	FIGO stage III (n = 3) serous ovarian cancer tissue samples	MALDI-MSI, PGC-LC–MS/MS	FFPE	Characterization of the spatial distribution of N-glycan structures within particular regions of the ovarian cancer sections (e.g., tumor, stroma, adipose tissue and necrotic areas). Detection of 40 individual N-glycan masses (including structural and compositional isomers). Delineation of cancerous and noncancerous tissue regions based solely on N-glycan structure distribution
Rémi Longuespée et al. [44]	Serous ovarian adenocarcinoma (n = 2), endometrioid ovarian adenocarcinoma (n = 2), and serous fallopian tube adenocarcinoma (n = 1) tissue samples	MALDI-MSI, LC-Orbitrap-MS	FFPE	Demonstration of a possible correlation between the serous ovarian adenocarcinoma and fallopian tubes (some biomarkers of ovarian cancer are actually fallopian tubes biomarkers). Proposing the origin of serous ovarian cancer as a consequence of metastasis from tumor cells derived from the fallopian tube
Oliver Klein et al. [34]	Low-grade serous ovarian carcinoma (n = 14), HGSOC (n = 19), serous borderline tumors (n = 14), ovarian clear-cell (n = 20) tissue samples	MALDI-MSI	FFPE	Demonstration that MALDI‐MSI combined with machine learning algorithms can classify different subtypes of epithelial ovarian cancer
Vivian Delcourt et al. [45]	Benign, tumor and necrotic/fibrotic regions of serous ovarian cancer biopsies (n = 18)	MALDI-MSI, nanoLC-MS/MS,	FF	Proposed approach might be useful for determination of protein changes in health and disease. Demonstration that 61 proteins are specific to the tumor region, 44 to the necrotic/fibrotic tumor region and 48 to the benign region
Marta Sans et al. [25]	Normal ovarian tissues (n = 15), borderline ovarian tumors (BOT) (n = 15), HGSOC (n = 48) tissue samples	DESI-MSI	FF	Identification of predictive markers of cancer aggressiveness, which involved various metabolites, free fatty acids, and complex lipids such as ceramides, cardiolipins, glycerophosphoglycerols, and glycerophosphocholines
Stephan Meding et al. [46]	Serous ovarian cancer (n = 31) tissue samples	MALDI-MSI, LC–MS/MS	FFPE	Detection of 3844 distinct peptide sequences (at a false discovery rate of 1%) in all samples (an average of 982 distinct peptide sequences per sample). Identification of a total of 840 proteins and, on average, 297 proteins per sample
Mohamed El Ayed et al. [20]	MALDI-MSI: FIGO stage III and stage IV (n = 48) ovarian cancer tissue samples derived from 25 patients, and benign tumors (n = 23) tissue samples NanoLC-ESI MS: grade III and IV ovarian cancer (n = 10) samples, and benign tumors (n = 10) samples	MALDI-MSI, nanoLC-MS/MS	FF	Detection of markers of ovarian carcinoma such as orosomucoid and lumican, which were highly glycosylated (consistent with the mucinous phenotype of ovarian cancers). Identification of two new biomarkers: fragment C-terminal of the PSME1 and mucin-9
Kristina Schwamborn et al. [47]	Serous ovarian carcinoma (n = 24) tissue samples, and samples from patients with non-ovarian carcinoma (n = 19, including gastric adenocarcinomas (n = 11), cholangiocarcinomas (n = 3), pancreatic adenocarcinomas (n = 2), lung adenocarcinomas (n = 2), and one ductal carcinoma of the breast (n = 1))	MALDI-MSI	FFPE	Demonstration that MALDI-MSI allows subtyping of malignant effusions to identify the origin of neoplastic cells. Identification of heat shock protein beta-1, tropomyosin, and cytokeratin-7 as significantly overexpressed in samples from serous ovarian carcinomas compared to other adenocarcinomas
Maria Luisa Dória et al. [24]	Normal ovary (n = 15) samples derived from 13 patients, normal fallopian tube (n = 6), malignant serous (n = 65), endometroid (n = 7), and clear cell (n = 6) ovarian cancer tissue samples	DESI-MSI	FF	Demonstration of the ability of the DESI-MSI technique to characterize ovarian cancer tissue samples while overcoming existing limitations in classical histopathology. Identification of molecular features (lipidomic profile) discriminating between studied tissue types
Endometrial cancer				
Parul Mittal et al. [40]	TMA: Endometrial cancer tumor metastasized to pelvic lymph nodes (with LNM) (n = 16), and without LNM (n = 27) LC–MS/MS and MALDI-MSI: Endometrial cancer tumor with LNM (n = 5) and without LNM (n = 5)	MALDI-MSI TMA, MALDI-MSI, IHC, nanoLC-MS/MS	FFPE	Demonstration that annexin A2 and α actinin 4 protein expression correlate with lymph node metastasis in endometrial cancer. Identification of m/z values which are associated with lymph node metastasis in endometrial cancer (by MALDI-MSI). Proving that MALDI-MSI shows higher accuracy than immunohistochemistry in predicting lymph node metastasis in endometrial cancer
Parul Mittal et al. [17]	Endometrial cancer tumor with LNM (n = 16) and without LNM (n = 27)	MALDI-MSI, LC–MS/MS	FFPE	Identification m/z values which can classify 88% of all tumors correctly (plectin and α-actin-2). These features may be used as potential markers for distinguishing endometrial cancer with and without LNM
Parul Mittal et al. [33]	Endometrial cancer tumor with LNM (n = 8) and without LNM (n = 20)	MALDI-MSI	FFPE	Demonstration that N-linked glycan may be useful for differentiate cancerous endometrium from normal, and endometrial cancer with LNM from endometrial cancer without LNM
Vulvar squamous cell carcinomas				
Chao Zhang, et al. [39]	MALDI-MSI: Vulvar squamous cell carcinoma (n = 6) tissue samples IHC: Vulvar squamous cell carcinoma (n = 8)	MALDI-MSI, IHC, nanoLC-MS/MS	FFPE	Providing an insight into the molecular profile of the vulvar intraepithelial neoplasia that seems to be more closely related to the healthy epithelium than the VSCC. Revealing decreased levels of Cytokeratin 5 in VSCC compared to the precursor lesion differentiated vulvar intraepithelial neoplasia

*DESI-MSI* desorption electrospray ionization mass spectrometry imaging; *FF* fresh frozen; *FFPE* formalin-fixed paraffin-embedded; *HGSOC* high-grade serous ovarian cancer; *IHC* immunohistochemistry; *LC‐MS* liquid chromatography-mass spectrometry; *LC-Orbitrap-MS* liquid chromatography-Orbitrap-mass spectrometry; *LNM* lymph node metastases; *MALDI-LTQ-Orbitrap-MS* matrix-assisted laser desorption ionization-linear ion quadrupole-orbitrap; *MALDI-MSI* matrix-assisted laser desorption ionization mass spectrometry imaging; *nanoLC-MS/MS* nano-liquid-chromatography tandem mass spectrometry; *PGC-LC–MS/MS* porous graphitic carbon liquid chromatography tandem mass spectrometry; *PSME1* Proteasome activator complex subunit 1; *TMA* tissue microarray; *VSCC* Vulvar squamous cell carcinoma

### Ovarian cancer

Ovarian cancer is one of the most fatal malignancies in adult women worldwide. The poor diagnosis comes from a lack of diagnostic markers for the early detection, rapid metastasis of the disease, and limited or modest understanding of the etiology. MSI has been widely applied in OC research. In recent years it has been successfully employed for spatial visualization of N-glycans in OC tumors [26, 43]. Glycoproteins are crucial mediators for cancer stromal interactions. Glycosylation takes part in cell recognition, cell–cell interactions, cell–cell communication, and adhesion [48]. Aberrant glycosylation can severely affect tumor cell biology, impacting all steps in tumor progression, from malignant transformation to metastasis. Zhang et al. in their study indicated that some major N-glycans are differentially distributed in tumors tissues and surrounding regions [42]. N-glycan Hex5HexNAc4 can be potentially involved in the interactions between cancer cells and stromal cells. Some glycans like Hex5HexNAc3dHex1 and Hex5HexNAc4NeuAc1 were abundant in the tumor proximal to stroma region. A low abundance of Hex5HexNAc4 was found in the distal stroma region. Briggs et al. investigated N-glycan distribution on OC tissue sections from early- and late-stage patients. Some complex neutral N-glycan structures, such as (Hex)2(HexNAc)2(Deoxyhexose)1 + (Man)3(GlcNAc)2 was observed only in the late-stage patients. Complex neutral N-glycans were more intense in the late-stage patients, however, fucosylated, truncated biantennary neutral N-glycan (Hex)2(HexNAc)2(Deoxyhexose)1 + (Man)3(GlcNAc)2 was only observed in the necrotic region which is not consistent with the observation in late-stage patients. In late-stage patients abundant N-glycans included oligomannose structure, (Hex) + (Man)3(GlcNAc)2, the complex neutral structure, (Hex)2(HexNAc)2 (Deoxyhexose)1 + (Man)3(GlcNAc)2. Everest-Dass et al. indicated that different glycan classes discriminated the four different tissues within the formalin-fixed paraffin-embedded (FFPE) section [43]. For necrotic region the most abundant was agalactosylated bi-antennary glycan (GlcNAc)2 + (Man)3(GlcNAc)2. In the stroma region complex fucosylated tri-antennary glycan ((Gal)3(GlcNAc)3(Fuc)1 + (Man)3(GlcNAc)2) was highly abundant. The pauci-mannose glycan (Man)3(GlcNAc)2 was observed predominantly in the adipose tissue. The high mannose glycan ((Man)5 + (Man)3(GlcNAc)2) was found in the tumor tissue. Increased endogenous sialylation might be one of the anti-apoptotic mechanisms in cancer cells [49]. Aberrant sialylation contributes to tumor growth and metastasis at multiple levels and it’s a key player in cancer progression [50]. Sialic acids might protect cells from apoptosis, enhancing invasion, and driving tumor growth. Sialidase and sialyltransferase enzymes expression is altered in cancer cells [51]. Sialidase expression is inversely associated with metastatic potential and tumor growth, probably through a regulation mechanism that suppresses cell growth and promotes apoptosis [49].

Longuespee et al. analyzed proteins from benign and three OC types including serous ovarian adenocarcinoma, endometrioid ovarian adenocarcinoma, and serous fallopian tube adenocarcinoma [44]. They discovered similarities between fallopian tube and serous ovarian adenocarcinoma. Two protein biomarkers were indicated as possibly engaged in the process of cell migration and metastasis: periostin and osteopontin. Periostin is known to be present in many different cancer cell types as it promotes cellular survival, angiogenesis, and resistance to hypoxia-induced cell death [52]. In cancer cells, periostin acts as a ligand for alpha-V/beta-3 and alpha-V/beta-5 integrins to support the adhesion and migration of epithelial cells. Periostin was highly expressed in fallopian tube cancer and not in OC cells. This study confirms the hypothesis that OC may begin as fallopian tube cancer. However, fallopian tube cancer and OC are considered the same disease. Periostin, which is expressed in fallopian tube cancer, may induce cell migration, and promote metastasis to host tissue. Osteopontin was proposed previously as an epithelial OC biomarker. It also promotes cell migration via integrin binding. Osteopontin takes part in critical processes for cancer progression such as immune response, cell adhesion and migration, and tumorigenesis [53].

In the study by Sans et al., they analyzed tissue samples from serous OCs using DESI-MSI. They observed the presence of different metabolic species such as small metabolites, saturated and unsaturated fatty acids, sphingolipids, ceramides, cardiolipins, glycerophosphoethanolamines, glycerophosphoglycerols, glycerophosphoserines, and glycerophosphoinositols [25]. A high abundance of ascorbic acid was observed in normal ovarian tissue. This is in line with previous studies pointing to the role of vitamin C in maintaining the proper functioning of the ovaries [54]. Gluconic acid was identified as a predictive marker for discrimination between HGSOC and borderline ovarian tumor [25]. It might be considered as a possible marker of OC aggressiveness. Two intermediates in the citric acid – succinate, and malate were also indicated as predictive markers of serous OCs. Succinate was reported to have oncogenic activity [55], while malate enhances fatty acid and cholesterol biosynthesis, enabling tumor growth [56]. Those results are in line with those published by Doria et al. where they used DESI-MSI to profile lipidome of different epithelial ovarian carcinomas, borderline ovarian tumors with normal ovarian stroma [24]. The most abundant were phosphatidylserine and phosphatidylethanolamine, phosphatidylglycerol and phosphatidylinositol in negative ion mode. In positive ion mode, the most abundant were phosphatidylcholine and phosphatidylethanolamine. Obtained results enabled building strong models to differentiate tissue types based on different lipid compositions of the analyzed tissues.

Schwamborn et al. in their study explored the role of MALDI-MSI to identify the precise origin of neoplastic cells in malignant effusions [47]. They analyzed cytology cell blocks from malignant effusions (serous ovarian carcinoma and several non-ovarian carcinomas including gastric adenocarcinoma). Serous OC samples were delineated with a sensitivity of 97.3% compared to gastric adenocarcinoma and with a sensitivity of 85.7% when compared to samples from all other included primary sites. Calculations were based on 195 tryptic peptides. Subtyping of malignant effusions using MALDI-MSI allowed to achieve similar results compared to currently used diagnostic methods. Taking into consideration progress in instrumentation and preparation protocols, analyzing samples with MALDI-MSI is comparable in terms of the time of the analyses.

Delcourt et al. analyzed HGSOC sections in terms of protein composition. Proteins identified in the benign regions were involved in the pathways implicated in cell growth, survival, adhesion, differentiation, and vascularization [45]. Proteins found in the necrotic/fibrotic regions are involved in inflammation, apoptosis, oxidative stress, and acute-phase reaction. Some proteins such as Cytokeratin 8 [57], Cofilin-1 [58], Utrophin [59], Cytokeratin 7 [60], Plasminogen activator inhibitor 1 RNA-binding protein [61] identified by Delcourt et al. were already known to be involved in OC. Proteins like gamma-synuclein, Lupus la protein, Nucleophosmin, Nuclease-sensitive element-binding protein 1, Probable ATP-dependent RNA helicase DDX17, and Hematological and neurological expressed 1-like protein were found in tumor and necrotic-fibrotic tumor regions. For benign and necrotic/fibrotic tumor regions salivary acidic proline-rich phosphoprotein ½ was found. Other proteins such as G antigen 7, High mobility group protein B1 (HMGB1), Glycogen synthase, G antigen 2B/2C, and Cilia- and flagella-associated protein 44 were specifically found only in the necrotic/fibrotic tumor region. Some of the identified proteins were studied previously as potentially involved in neoplastic processes. Homeodomain-interacting protein kinase 1 was suggested in a previous study as a modulator of p53 activity that can promote oncogenesis [62]. Identified Protein S100A11 was previously found to be upregulated in human cancer tissues promoting growth, invasion, and migrations of cancer cells [63]. The potential role of Nucleophosmin is associated with increased cell growth, proliferation, and the inhibition of differentiation and apoptosis [64]. Overexpressed gamma-synuclein compromises normal mitotic checkpoint controls, resulting in multinucleation and faster cell growth [65]. HMGB1 was previously indicated as a novel biomarker of OC. Overexpression of HMGB1 was associated with poor clinicopathologic features [66]. What is more interesting both HMGB1 and Macrophage migration inhibition factor increased levels were associated with higher plasma levels of metalloproteinase-9 which was indicated as a potential prognostic factor for OC [67].

Our group analyzed two ovarian tumors (low-grade serous borderline ovarian tumor and ovarian fibrothecoma) often being a diagnostic challenge. Some characteristic m/z values for each tissue were shown using the MALDI-MSI technique. Although they were not able to identify differentiating peaks, they proved the huge potential of MALDI-MSI in OC research by showing a close correlation of the molecular maps with the morphological and histopathological features of the tissue which allowed the identification of different tissue types within the same tissue sample [41].

Machine learning is an interesting approach in biomarker research and has great potential when combined with MALDI-MSI. Kassuhn et al. used machine learning and MALDI-MSI for the classification of molecular subtypes of HGSOC. The applied novel strategy allowed for near-perfect stroma classification. One of the most discriminative compounds for malignancy was a peptide linked to histone H1.2 specifically expressed in malignant tissue [27]. Klein et al. applied MALDI-MSI with machine learning for the classification of epithelial OC histotypes. Four different histological types of epithelial OC were included in a tissue microarray analysis. They investigated the potential of different machine learning methods for classification strategies using MALDI-MSI proteomic data. The best histotype prediction was achieved by using a convolutional neural network [34]. The result of this study proved that MALDI-MSI combined with machine learning approaches can be a valuable diagnostic tool for the differentiation of epithelial OC histotypes.

### Endometrial cancer

Endometrial cancer (EC) is a malignant tumor that originates in the inner lining of the uterus. It is the most common gynecologic malignancy in developed countries. Currently, the diagnosis of endometrial cancer is based on the histopathological assessment of endometrial tissue. This diagnostic method in up to 50% of cases leads to the incorrect assignment of grade and histological type to the neoplasm [5]. Patients with the EC diagnosis at an early stage, with cancer still being confined to the uterus, have a 5-year survival rate of 95% [68]. Detection of the EC, when cancer has metastasized, lowers the survival rate down to 16% [69]. First-line treatment of endometrial cancer is based on surgery. The extent of the surgical approach depends on histological type, grade, and stage. Many patients will undergo pelvic lymphadenectomy to precisely define the stage and tailor adjuvant therapy. The procedure itself can cause many health problems including lower extremity lymphoedema. There is a clear need to focus on predictive markers of lymph node metastases (LNM), which could significantly reduce the patient’s exposure to unnecessary surgeries, as well as the associated health complications. Tissue imaging mass spectrometry MALDI-MSI has been implemented to study endometrial cancer markers. In recent years a few articles about using the MALDI-MSI technique in EC research were published. MALDI-MSI demonstrated potential in discriminating regions of normal endometrium from cancer [17, 33, 40]. Moreover, Mittal et al. were able to predict the LNM status of EC patients from primary tumor cores with an accuracy of 88% [17]. Also, 2 proteins—α-Actin-2 and plectin were identified and validated as potential discriminators for LNM in endometrial cancer. Mittal et al. in their research analyzed available data published so far and chose 60 proteins having differential potential between patients with ECs with LNM and those without LNM. They were able to detect 23 of those proteins using the LC–ESI–MS/MS method. 5 of those proteins were observed with the differential abundance. The visualization of the differentially expressed proteins using MALDI-MSI was performed using the whole tissue section as well as tissue microarrays. Identified proteins were further validated by immunohistochemistry. Three proteins were selected as those with differential staining. Annexin A2 was one that was upregulated, while annexin A1 and α actinin 4 were downregulated in tumors with LNM in comparison to those without LNM. A predictive model based on MALDI-MSI analysis showed higher prognostic accuracy than the model based on IHC analysis. It’s the first study showing low expression of actinin 4 in primary tumors with LNM compared to those without LNM. The analysis of MALDI-MSI data with machine learning techniques enhanced the capacity to predict LNM and may improve the stratification of EC patients [12]. Glycosylation is one of the most common post-translational modifications of human proteins. Previously, it has been shown that a lower abundance of N-linked glycans is correlated with metastatic disease [70]. In line with these discoveries, Mittal et al. showed in their next study that a low abundance of a single complex core-fucosylated N-linked glycan is observed in the primary tumor tissue with LNM compared to patients without LNM [33]. They also observed a higher abundance of oligomannose glycans in tumor compared to normal regions and a lower abundance of complex N-linked glycans. These observations are in line with previous studies but in different cancers [12]. However, due to the low number of patients in those studies, further work on a larger patient cohort is needed. MALDI MSI demonstrates promising results and supports the need to study the role of N-linked glycans so the altered N-glycosylation may be used to stratify EC patients.

### Vulvar squamous cell carcinomas

Only one study by Zhang et al. [39] focused on implementing MALDI-MSI in vulvar squamous cell carcinoma (VSCC) to analyze tissue sections containing regions of healthy vulvar epithelium, differentiated vulvar intraepithelial neoplasia (dVIN), and VSCC. The nanoflow liquid chromatography-tandem mass spectrometry (nano-LC–MS/MS) was applied to identify proteins selected by MALDI-MSI. Further IHC analysis revealed an increased level of Cytokeratin 5 in VSCC compared to dVIN and healthy tissue. No significant difference was observed when comparing dVIN and healthy epithelium.

## MSI challenges

Though MSI is a very promising diagnostic and research platform, certain technical limitations make it challenging to apply it in routine clinical practice. The presented work comprises a concise review of ongoing studies on gynecological cancers. Not to diminish the importance of described before findings, it should be also mentioned that numerous issues were mentioned by authors as limitations of studies and these challenges should be consider with respect to results from MSI-based studies. It is especially important when drawing conclusions, comparing different studies, or designing future projects. The limitations described below comprise the challenging issues mentioned by the authors of reviewed articles or being directly related to studies included in this review but is not an exhaustive view of all methodological limitations affected by MSI-based research.

### Instrument limitations

Instruments dedicated to performing MSI experiments have their own limitations. In general, Mittal et al. [17] highlighted the difficulties related to obtaining direct identification of on-tissue peptides based only on a single MALDI-MSI experiment, despite using relatively high mass accuracy. It is impossible to detect all classes of chemical compounds in a single experiment. To sum up, mass spectrometers have restrictions in the mass range, resolution, type of *m/z* detection, and scanning time. These features have a direct impact on the possible results to obtain, the same limiting e.g., the spectrum of analytes or even selectivity.

Obtaining satisfactory ionization and sensitive detection of a wide mass range of compounds is another instrument constraint. Some authors emphasize the difficulties in imaging of e.g., glycans. Everest-Dass et al. stress the need to further optimize the sensitivity of detection for N-glycan in MALDI-MSI experiments [43]. The other issue might be the high hydrophilicity of glycans and various possible isomeric structural forms that hinder direct and reliable detection [71]. Zhang et al. indicate a necessity of derivatization and/or chemical modification of glycans prior to analysis [42]. Quite a similar dilemma related to many isobaric ions was mentioned by Kulbe et al. [72]. The presence of so-called chimera spectra impairs the direct identification of interesting *m/z* values. That indicates another ‘demanding step’ to obtain reliable MSI-based results—compound identification, which is also directly related to e.g., data processing (see below the section ‘Data processing’).

Another issue directly related to instrumentation, and specifically to detection was mentioned by Delcourt et al. [45]. The authors mentioned two alternative approaches in proteomics: the top-down and the shotgun strategy. Both are firmly established in analytics, but each of these strategies has its own limitation. In the context of small proteins, which are the object of interest in Delcourt’s study, the authors decided to choose shotgun proteomics for their detection. The decisive factor was a low amount of generated tryptic peptides and the generally fewer presence of enzyme cleavage sites which potentially could decrease identification. Thus, strategy chosen for identifying proteins might directly affect the obtained results and should be thoroughly considered at the stage of planning the experiment, with special emphasis on the characteristics of the proteins/peptides of interest.

The importance of MS sensitivity is essential for MSI studies due to its direct impact on the detected feature sets selected for further processing. As it was mentioned by Kassuhn et al. even decimal mass deviation can change selected feature sets between experiments and between laboratories, affecting both reproducibility and reliability of multicenter studies [27].

Using an axial MALDI-TOF entails occurring inaccuracies in measuring a molecule’s mass due to the instruments’ precision and initial velocity. To deal with these inaccuracies, usually, mass calibration is performed. Two methods are most often chosen for mass calibration: internal or external calibration. Thus, a relative mass alignment might lead to some discrepancies in the results. Some authors propose its own method for mass calibration [73]. On one hand, it can broaden the spectrum of available methods and possibly contribute to obtaining more precise and accurate mass detection from tissue. On the other hand, it leads to the necessity of more thoughtful planning of the study to eliminate discrepancies in multilibrary studies or between-laboratory protocols inconsistency.

Another limitation listed by the authors of reviewed studies was the spatial resolution and mass resolution of the MSI experiment. Kawashima et al. [74] concluded that the current MALDI-MSI resolution is too low for reliable analysis of the lipid profiling of single cells. Additionally, the authors listed such limiting factors of MALDI-MSI as limited lipid species coverage in a single experiment and the quality of the image affected by sample processing.

The literature shows that various raster widths were used e.g. 50 µm [27, 34, 72], 100 µm [33, 42], 200 µm [41]. These sizes being close to the size of eucaryotic cells are still not enough for reliable single cell imaging.

### Reproducibility and lack of the multicenter studies

Next, a very common limiting factor affecting the reproducibility in reviewed studies is the sparsity of diagnostic material because of the limited number of patients. The current studies often are performed on very few numbers of real samples [26, 41]. As far as it is still interesting as a proof-of-concept study and for identifying marker candidates, such a group size is not sufficient for proper and reliable clinical validation [72]. The limited number of samples in MSI studies is a multifactorial issue. First, due to the invasiveness of the sample acquisition procedure, there is a limited number of samples that can be included in the study. Further, it might be the difficulties with the selection of an appropriate control sample/control group to compare the observed alteration in the proteome. On the one hand, the analysis of healthy tissue from the same patient might be the most interesting comparison, but the invasiveness of the tissue section makes it impossible for a non-invasive analysis of healthy tissue. The need for proper control group was highlighted e.g., by Pietkiewicz et al. [41] and [43]. Then, on the other hand, to find a universal marker and definitively validate the hypotheses there is a need for wide, multicenter studies with a large cohort of patients. It was summarized by Longuespee et al. by underlying the need for repetition of the experiment in a large cohort of tissues with keeping in mind the importance of the issue of inter-patient variability but also highlighting the significance of the same patient for comparative analysis as a perfect internal control for the experiment—MSI analysis with their ‘anatomical context’ [44]. Additionally, the team of Klein suggested that the limited number of samples can also lead to some statistical complications, which could potentially be eliminated by increased by the larger numbers of tissues included in the study [34]. The need for a follow-up study with an evaluation of more clinically relevant cases was mentioned also by Sans et al. [25] and Mittal [33]. Despite the fact that MSI studies enable to evaluate of the spatial distribution of hundreds of features within one single experiment surpassing the limitations of staining techniques [47], the proper control and sparsity of diagnostic material still are one of the major problems that limit the repeatability of research.

### Protocols inconsistency and variance in sample preparation protocols

Repeatable study protocols and sample preparation might be crucial for drawing reliable conclusions from MSI-based research. Unfortunately, for now, there are a variety of approaches for sample preparation and study design leading to potential discrepancies in the results.

Considering MSI experiments related to clinical diagnostic, tissue preparation might be a reason for some discrepancies between studies. Various issues related e.g., tissue thickness, method of cryosectioning, incubation time, drying time, and the temperature during the tissue preparation should be considered during MSI-based study design as well as comparing results of different studies. A review of literature on gynecological cancers clearly shows the differences in these pre-analytical steps—samples were prepared in different ways. A simpler approach was based on the collection of samples during the surgery, frozen, and stored at − 80 °C, followed by cutting the tissues into thin slices and mounted on electrically conductive sample slices [20]. Using fresh frozen tissues might be performed using various protocols (a different condition during each step). Alternatively, more steps are necessary to use tissue microarrays (TMA). This approach might base on the preparation of tissue cores on paraffin blocks by cutting them from e.g., FFPE tissue samples. TMA is described to be less time-consuming and less prone to technical variation related to the preparation of each tissue section individually. Thus, TMA seems to be ideal for large patient cohorts screening based on MSI, due to the fact of the possibility of comprising dozens of tissue cores in one paraffin block [46]. Both approaches require a different method of sample mounting on a slide glass and thus the implementation of additional procedures including using a cryopreservation medium or deparaffinization. Moreover, some authors underlined the preservation of tissue integrity and the need for avoiding molecular composition changes during sample preparation steps [44].

Among works included in this review most often used technique was MALDI MSI (Table 1), despite the type of cancer it was the most often used ionization method. The use of MALDI as an ionization method entails the necessity of using the matrix as a factor that contributes to ionization. Across reviewed studies, authors decided to use various matrices such as cyano-4-hydroxycinnamic acid [17, 20, 33, 34, 39–41, 44, 47, 72, 73, 75, 76]; 2,5-Dihydroxybenzoic acid [26, 43, 45, 46] and sinapinic acid [20]. The identification of a suitable matrix for MALDI-based experiments plays a crucial role in the possibility of ionization and further detection of an appropriate group of biomolecules. The same, as was mentioned by the Longuespée et al. matrix choice is one of the essential points for a successful MALDI experiment [44]. However, it is also a cause of variability in the results of MSI-based studies. It also stays an indispensable part of the MALDI methodology, and until the universal matrix for wide spectrum compounds will be discovered, there is a need for matrix selection adopted for the appropriate application.

The next step of the MALDI-MSI experiment is also a possible cause of some changeability. This step is on-tissue matrix deposition. The method of matrix application might be a source of different results e.g., homogeneity of the matrix layer, and size of the matrix crystals affecting the results. The reviewed studies have also been based on different equipment used for matrix deposition. The authors often decided to use a commercial available equipment [26, 34, 39, 40, 73, 75], but also performed a matrix deposition through manually spraying [45]. Bearing in mind the role of the matrix in MALDI experiments, its deposition method it is nevertheless important. The fact of discrepancies in MALDI MSI studies resulting from matrix deposition methods, equipment used, applying protocol, type and quality of the tissue or the matrix used should be considered and carefully optimized.

Longuespée et al. underline also the fact of possible analyte delocalization across the tissue sample during pre-analytical steps [44]. It might be a reason for possible bias in MSI studies and precautionary steps should be taken into account to eliminate that inconsistency factors during matrix deposition as well as other pre-analytical sample preparation steps.

Differences in data acquisition parameters might result in different features detection. Data acquisition depends on the mass analyzer, software used, and acquisition parameters which might be different in different studies. These parameters e.g., mass range, the type of data required (accurate or nominal mass), ion source voltages, temperature, ionization technique and polarity (negative or positive ion mode), peptide charge, or the number of spectra acquired per sample are directly related with compounds of interest intended to detection. However, these variations might lead to differences in detected m/z features and thus differences in identified molecules. During this step, it is easy to arbitrarily exclude some potentially interesting compounds through the too strict acquisition criteria. The same, data acquisition parameters selection step might be crucial for all further parts of the experiment such as detection, identification, statistical testing, and the significant compound selection e.g., biochemical interpretation.

Table 1 showed that for the biomarker discovery approach MALDI MSI method seems to be the method of choice, most often selected by the authors. However, it should be also noted that other instruments might be successfully used for imaging studies. DESI MSI might be found as a competitive tool for histological studies, e.g., due to fact of lack of use of ionization matrices. This fact eliminates some steps during sample preparation, and thus decreases variance from matrix peaks as well as completely eliminates matrix-related issues during sample preparation [24].

The review of the MSI-based studies has revealed the entire spectrum of differences in the protocols applied during sample preparation. Different tissue sectioning and preparation, and various sample preparation adapted to the MS technique were used (e.g. MALDI matrices and matrix deposition methods) [20, 41]. That variations are very often obligate and related directly to the instrument used. That leads us to the conclusion that some protocols or sample preparation inconsistencies are inseparable from instrumentation differences. It is known that the compounds of interest may require to use of completely different instruments, equipment, or e.g. derivatization prior to the analysis [26, 42, 43]. On the other hand, using the analogous instrument researchers may focus on e.g. different molecules, and types of samples or have different study hypotheses and in the results, they have to modify protocols or sample preparation for a specific application [27, 47].

What is worth highlighting some authors indicate that different signal intensity may be also related to the tissue type? According to the authors’ hypothesis, some measurement artifacts may cause a variance in signal intensity for different tissues. Hernadez et al. proposed to undertake some corrective actions to minimize that impact (e.g. via relative intensity thresholding) [75].

Protocol inconsistency remains an open issue. For now, it is impossible to fully standardize the sample preparation when different instruments are used. The open science idea, raw data repositories, and preparing the database with a full description of the all steps performed in experiments might be helpful to unify the MSI-based protocols. Nevertheless, to be the most reliable, the impact of mentioned differences should be considered and taken into account when comparing data from different research labs.

### Data processing

MSI techniques with their biggest advantage of identifying spatial distribution of analytes directly on tissue, generate complex and high-dimensional data. It requires the use of thoughtful and repeatable bioinformatics techniques for raw data computation. It is especially important due to differences in the quality of data acquired, pre-processing methods, and data processing.

The first issue is related to the type of data acquired. Different techniques and instrumentation used for MSI experiments such as DESI-MS [25] MALDI-LTQ-Orbitrap [42] or MALDI-MSI [27] give various raw data which might differ not only on such parameters as spatial resolution or size of the image but also with the quality of the registered data. It dictates the necessity of proper data processing, not always analogous between different instruments. Thereby, the type of mass spectrometer used obligates not only e.g., mass range (type of analytes of interest) but also the proper data processing.

The pre-processing process might be multistep and various procedures such as a signal-to-noise ratio for peaks detection selection, baseline subtraction, and smoothing or normalization might be applied to prepare the obtained raw MS spectrum for further analysis. Different procedures and parameters are used by the authors for this process [39, 43, 45].

The results of experiments might be also dictated by the identification strategy applied. First of all, it should be highlighted that analytes identification in MSI-based studies might require an additional step or even additional experiments for *m/z* feature identification e.g., by ESI–MS/MS experiment [43]. Thus, some discrepancies depending on the analytical technique selected for the research, and further differences in data processing effect the results when comparing different studies. Some parameters such as mass tolerance, expected score (E-value), peptide false discovery rate, database type, and version could affect the number of identified features [39, 45], reaching even hundreds of proteins per sample [46].

The obtained MS data include both molecular and spatial information and require proper statistical evaluation to find an alteration in molecular expression between compared groups and to build accurate and reliable statistical classifiers which could be useful to build tissue-based molecular classifiers [25]. The analysis of possible statistical approaches goes beyond the interest of this work, however, it should be noted that the authors used different approaches to typing significant statistical changes. Thus, comparing the results of the different studies the applied statistical approach should be thoroughly analyzed with special emphasis put on e.g., the size group, type of data used, and study design (despite the fact that MSI-based research was intended to be qualitative through the determining the spatial distribution of compounds of interest with their relative intensities, some approaches or hyphenated techniques enable to obtain quantification [42]).

### Other limitations

The last challenge which should be listed is the lack of knowledge about the role of discovered *m/z* features, proteins, peptides, compounds, and their role in biology. Some of the authors toward scientific transparency directly say that the function of some molecules remains unknown [45]. Thus, the accurate interpretation of the biological functions of the compounds of interest is a challenge for all mass spectrometry-based discovery research, not only MSI studies. In our opinion, the biochemical and physiological interpretation of the role of founded molecules is and will remain the greatest challenge for researchers. To overcome the challenges we are facing, there is a need for standardized, multiomic studies comprising discoveries from various omics coupled with in-depth biochemical interpretation.

The above-mentioned limitations summarized in our review of MSI-based research on gynecological cancers are not all challenging issues related to tissue imaging, which should be clarified and standardized to make MSI a widely used and reliable diagnostic method. However, the undoubted advantages of MSI, outweigh the current diagnostic methods, reinforcing the necessity of further research and development of this technique. In our opinion, overcoming these challenges will revolutionize the future of medical diagnostics.

### Muticenter studies of clinical samples

Clinical studies require many biological replicates. One of the ways to achieve this is by combining the findings from different research centers. Even though this kind of approach might be affected by potential “location” and “time” factors, it has the potential to identify real biological differences. A common data format has been established for MSI (imzML), guidelines specifying the data and metadata that should be collected have been developed [77], and the data repositories have been established. A study taking into consideration not only MSI instrumentation, but also different preparation protocols have been performed. The imzML conversion allows exchanging the fully functional MS imaging datasets between the different laboratories even with the variety of mass spectrometers used for this study. Taken together multicenter studies approach, guidelines, data format, and data repository can improve the reliability and transparency of MSI studies.

## MSI perspectives

MSI is a powerful tool for untargeted analyses of the spatial distribution of different biomolecules in a variety of samples. In recent years the technological advancement in the field of MSI has been significant. Improvements in reproducible sample preparation protocols and data interpretation, instruments with higher acquisition speeds, and enhanced spatial resolution. Moreover, new methods and techniques have been presented that may have a huge impact on the further use and development of this promising technique. Developments described in the following subsections indicate further directions in the MSI field creating potential new paths of development of this increasingly well-established tool in clinical practice and the pharmaceutical industry.

### Imaging mass cytometry (IMC)

Previously, mass cytometry was only used to analyze cells in suspension, so the information about cell–cell interactions within tumor tissue/microenvironment was lost. IMC is a high-dimensional technique that provides an integrative spatial tissue analysis and enables imaging of up to 40 protein markers on both cellular and tissue levels. This technique combines laser-ablation-inductively coupled plasma mass spectrometry (LA-ICPMS) with the resolution of 1 µm and the cytometry by time-of-flight [78]. Targets are labeled with metal-tagged antibodies. The main advantage of this method is that compared to fluorescent microscopy there is almost no noise in the data as each metal isotope has its own detection peak and does not overlap with other metal isotopes. What is more, there is almost zero background noise so the contrast between markers of interest and the background is perfect for image analysis. Because this IMC technique allows for upward of 40 markers to be simultaneously stained, acquired, and visualized from a single slide. The main limitations of this technique are the availability and cost of the antibodies. It is likely that thanks to the multiplex capabilities and improved quantitation and its easy integration into clinical practice will lead to the rapid development of multiplex and novel biomarker panels, but only the clinical impact of these biomarker panels will prove the usefulness of IMC as a routine clinical tool. In the study by Jackson et al., it has been highlighted that single-cell pathology can better classify patients with a specific clinical outcome than currently used clinical subtyping of breast cancer [79]. Taken together, the IMC technique provides much more high-quality information crucial for identifying new biomarkers.

### Multiplexed ion beam imaging (MIBI)

It is a technique designed for multiplexing based on SIMS. In this method, tissue is immunoprobed with metal-tagged antibodies and then it is analyzed using TOF secondary ion mass spectrometry. Based on the unique metal isotope label of each antibody, the detected compounds are assigned to target molecules. MIBI allows analyzing up to 100 targets simultaneously measuring protein levels on individual cells, providing information about cell morphology and localization. This technique has many advantages over conventional IHC techniques. Five-log dynamic range exceeds that of chromogenic IHC and immunofluorescence, there is no background signal due to autofluorescence, no spectral overlap is observed because the mass accuracy can resolve less than a fraction of a dalton at the low resolution, the interferations of residual isotopic contaminants or metal oxide abducts with the reporter masses are not observed [80]. Also, the assay’s linearity is better than IHC chromogenic and immunofluorescence because secondary labeling or amplified detection is required. This technique provides the ability for understanding the biological mechanisms in the tumor microenvironment. Based on the acquired images cell boundaries can be defined, cells might be classified into cell class, such as nonproliferating or proliferating tumor cells. Only a few studies focused on MIBI in breast cancer and no studies that discussed MIBI in gynecological cancers were found. In the study by Rost et al., the quantitation of human epidermal growth factor receptor 2 (HER2) expression was performed in breast cancer tissue with known HER2 status [81]. The strong correlation between IHC scores determined by pathologists and quantitation of HER2 by MIBI was identified. According to the study by Keren et al. the MIBI method allowed for describing the connection between cell phenotype and tissue structure in triple-negative breast cancer [82]. They found out that the tumor-immune microenvironment is divided into prototypical archetypes with structured cell composition, spatial arrangement, and expression of regulatory proteins that are involved in overall survival. The application of MIBI showed that tumor expression and immune composition are interrelated within a histological context.

### International validation

Taking into consideration all advances in the field of applying mass spectrometry imaging in clinical research it is obvious that it can become a valuable clinical technique applied worldwide. However, to follow the path of MALDI MS Biotyper and iKnife as internationally recognized tools that are routinely used to classify complex cell structures or to classify tissues in situ during surgery respectively, MSI still needs time and refinement. MSI technique provides many applications and each of them has the potential to become transformative. In-situ tissue classification, searching for new prognostic, predictive, and diagnostic biomarkers, ability to image different compound classes like metabolites, lipids, proteins, and drugs are the applications that with standardized methods and large-scale multicenter validation studies will prove their clinical utility and significance. With the rapid expansion of MSI, there is a need for a uniform standard for reproducibility between users, laboratories, and biological samples [83].

### Three-dimensional (3D) MSI

3D MSI is commonly done through serial sectioning of a sample. Each tissue section is analyzed in two-dimensional (2D) and then reconstruction of a 3D model is performed. However, this approach cannot be used for samples that cannot be sectioned such as 3D cell cultures. Also, reconstruction of 2D MSI can be challenging with image alignment and registration as samples may not lay flat on the slide or may have density differences. In recent years topography-integrated MSI instrumentations have been developed such as constant-distance mode nanospray desorption electrospray ionization mass spectrometry imaging [84] or custom-built laser ablation electrospray ionization source to accommodate the topography of non-flat sample surfaces [85]. The idea of “depth profiling” can be very useful and utilized in cancer research. Some developments in recent years such as femtosecond laser ionization source for imaging with a 7 µm depth resolution, submicrometer depth resolution, down to 20 nm, using extreme ultraviolet laser light prove the huge potential in 3D imaging and show its future application in more complex cancer systems.

### MALDI-IHC

One of the newest and most interesting methods is MALDI-IHC proposed by Yagnik et al. [86]. It enables highly multiplexed IHC based on MALDI-MSI thanks to novel photocleavable mass tags (PC-MTs) for easy antibody labeling. PC-MTs are modified polypeptides consisting of a mass reporter region, a high-performance photo-cleavage linker (PC-Linker) incorporated into a peptide via solid-phase synthesis, and an N-hydroxysuccinimide probe reactive moiety near the C-terminal. The probes are manufactured in a one-step reaction. The modern PC-Linker ensures high sensitivity allowing the analysis of a wide spectrum of biomarkers in various tissues. In heterogeneous tissues, it is important to simultaneously determine the localization of a number of biomarkers. Fluorescence microscopy is limited to the simultaneous detection of only a few biomarkers because of the broad excitation and emission bands of fluorophores that may cause spectral overlap [87]. In contrast, the label-free untargeted MSI technique can directly analyze lipids, metabolites, and drugs which is not achievable using standard IHC or imaging mass cytometry methods. The versatility of this method allows the performance of label-free untargeted MSI analyses of small molecules as well as targeted MSI analyses of macromolecular biomarkers based on PC-MTs. MALDI-IHC method can provide a new dimension to tumor-tissue specimens analyses and consequently obtain improved therapy and patient outcomes. Also, this could be a step forward for researchers. Mentioned advances will enable the investigation of the spatial distribution of hundreds of biomolecules at the cellular level in different fields e.g., proteomics, tissue pathology tissue diagnostics, therapeutics, and personalized medicine.

## Future clinical applications in gynecologic oncology

MSI has the potential to enhance future clinical decisions in gynecologic oncology. It may improve diagnostic procedures, inform on prognosis and indicate better management options. Information acquired by patients’ examination, routine laboratory, and imaging studies often remains insufficient to choose the most optimal treatment. High-throughput techniques, such as MSI, yield a multitude of data that may improve the decision-making process in patients’ care. On top of tissue content, MSI brings an additional dimension thanks to the analysis of spatial relationships. The context of the microenvironment, including immune infiltration and aspect of the surrounding stroma, can be measured by MSI. Those features are often considered qualitatively by a pathologist at the time of tumor diagnosis but are rarely used at further management stages. Thanks to MSI, many spatial features of cancer tissue combined with protein, lipid, or small molecule content may constitute a highly informative multidimensional input for decision algorithms.

MSI may make the initial pathological diagnosis more precise. Identifying ovarian cancer subtypes can be challenging, especially in poorly differentiated tumors. The maintenance therapy, however, varies for distinct types. For instance, PARP inhibitors are mainly applicable in high-grade serous and endometroid but not mucinous tumors. There are clinical trials designed separately for the mucinous subtype. The management will also differ for borderline and low-grade ovarian cancers—the first will require only observation; the latter will benefit from hormonal treatment reducing exposure to estrogens. The distinction between those two types might be a challenge. Though the diagnosis of the subtype and grade might be evident for an experienced pathologist, there are samples with mixed cancer types or unclear morphological and staining features. MSI has the potential to help the pathologist in making diagnostic decisions based on more quantitative criteria.

Patterns defined by MSI could provide more accurate information on a patient's prognosis. Certain studies have already demonstrated that MSI may improve the detection of lymph node metastasis in endometrial cancer without needing lymph node resection [17, 33]. Identifying patients at risk of distant foci is crucial because they will require potentially more extended surgery, including lymphadenectomy. Given the potentially severe side effects of unnecessary lymphadenectomy, estimating the risk of disseminated disease by the MSI analysis of primary tumor is especially promising. Identifying those patients is also essential as lymph node metastasis defines the advanced stage of disease (FIGO III) that requires further chemotherapy and radiation therapy. Patients not receiving that treatment are at higher risk of endometrial cancer relapse with a poor prognosis. Hence, developing MSI-based algorithms may enhance determining prognosis and tumor staging without the need for extensive surgery and optimize the choice of subsequent therapy.

MSI might be clinically applicable also in predicting response to therapy in ovarian cancer. Identification of responders to PARP inhibitors among OC patients could be one of the future clinical applications of MSI. In recent years, this maintenance therapy significantly improved OC patients’ survival and is primarily effective in tumors with homologous recombination deficit (HRD) [88, 89]. Though HRD is mainly measured by genome analysis [90], disturbances in HRD pathways or the emergence of highly aberrant new proteins might be a hallmark of this process as measured by MSI. More importantly, MSI analysis focused on non-responders might indicate mechanisms that contribute to resistance to PARPi. Based on recent clinical trials, some patients will benefit from a combination therapy of PARPi and antiangiogenic treatment [91]. It remains unclear how to choose PARPi monotherapy as compared to this combination. It is possible that, thanks to current research, MSI will help in this clinical decision in the future. Another clinically relevant area is the management of patients affected with platinum-resistant recurrent OC, who have limited treatment options [92]. Some of them can be candidates for biomarker-directed clinical trials, for instance, with an antibody against folate receptor alpha [93, 94]. It is possible that MSI will identify those quickly progressive tumors even prior to the onset of the platinum-based first-line treatment. The recurrent tumors have been well characterized in terms of genome aberrations [95]. Applying an alternative technique, such as MSI, in identifying new targets, including neoantigens, could advance treatment strategies. Many patients with OC have familial syndromes predisposing to malignancy, such as BRCA1/2 mutation [96]. Some of those patients will have a clear family history of OC with no genomic alterations identified. Future MSI research could focus on searching image patterns linked to the disease’s familial background. MSI might also apply to the analysis of cell clusters isolated from ascites fluid at the initial OC diagnosis. However, this approach would require a better resolution adjusted to the cell cluster size.

MSI might bring new solutions in predicting response to treatment in patients with endometrial cancer. Defining subtypes of endometrial cancer (microsatellite instability, high-copy number, low-copy number, and mutant POLE types) in The Cancer Genome Atlas (TCGA) demonstrated clinical utility [4]. For instance, patients assigned to the mismatch-repair deficiency (dMMR, microsatellite instability) class can benefit from immunotherapy based on pembrolizumab or dosterlimab in cases of advanced or recurrent disease [97]. Currently, an assignment to the class is based on the combination of immunohistochemistry and targeted next-generation sequencing. Phenotyping based on MSI could reveal new subtypes or improve the precise identification of already defined groups, following examples of successful classification based on pathology image analysis [98]. Therefore, MSI may help to indicate responders to immunotherapy among patients with endometrial cancer.

## Concluding remarks

MSI has expanded in recent years and due to its wide variety of applications is slowly being introduced into commercial pathology laboratories around the world both as a complement and replacement to other imaging methods. The introduction of MSI with its current potential to extend diagnostic and prognostic capabilities can revolutionize clinical pathology. Considering that this technique presents an urgent need for improvements in reproducibility in sample preparation, data processing, and some instrumentation limitations the full potential of MSI is yet to be demonstrated.

## Data Availability

The data used to support the findings of this study are included within the article.

## Abbreviations

2D: Two-dimensional
3D: Three-dimensional
CA125: Carcinoma antigen 125
DESI: Desorption electrospray ionization
dVIN: Differentiated vulvar intraepithelial neoplasia
EC: Endometrial cancer
FDA: Food and Drug Administration
FFPE: Formalin-fixed paraffin-embedded
FISH: Fluorescence in situ hybridization
HER2: Human epidermal growth factor receptor 2
HGSOC: High-grade serous ovarian cancer
HRD: Homologous recombination deficit
IHC: Immunohistochemistry
IMC: Imaging mass cytometry
IVDMIA: In vitro Diagnostic multivariate index assay
LA–ICPMS: Laser-ablation–inductively coupled plasma mass spectrometry
LC-ESI–MS/MS: Liquid-chromatography electrospray ionization tandem mass spectrometry
LNM: Lymph node metastases
MALDI: Matrix-assisted laser desorption ionization
MALDI-MSI: Matrix-assisted laser desorption ionization mass spectrometry imaging
MALDESI: Matrix-assisted laser desorption electrospray ionization
MALDI-LTQ-Orbitrap: Matrix-assisted laser desorption ionization-linear ion quadrupole-orbitrap
MeSH: Medical subject headings
MIBI: Multiplexed ion beam imaging
MS: Mass spectrometry
MSI: Mass spectrometry imaging
Nano-LC–MS/MS: Nano-liquid–chromatography tandem mass spectrometry
OC: Ovarian cancer
PARPi: Poly (adp-ribose) polymerase inhibitors
PC-MTs: Photocleavable mass tags
PC-Linker: Photo-cleavage linker
SIMS: Secondary ion mass spectrometry
TCGA: The cancer genome atlas
TOF: Time of flight
TMA: Tissue microarrays
VSCC: Vulvar squamous cell carcinoma
